# Optimality Principles in Human Point-to-Manifold Reaching Accounting for Muscle Dynamics

**DOI:** 10.3389/fncom.2020.00038

**Published:** 2020-05-15

**Authors:** Isabell Wochner, Danny Driess, Heiko Zimmermann, Daniel F. B. Haeufle, Marc Toussaint, Syn Schmitt

**Affiliations:** ^1^Institute for Modelling and Simulation of Biomechanical Systems, University of Stuttgart, Stuttgart, Germany; ^2^Machine Learning and Robotics Lab, University of Stuttgart, Stuttgart, Germany; ^3^Khoury College of Computer Sciences, Northeastern University, Boston, MA, United States; ^4^Hertie Institute for Clinical Brain Research, and Werner Reichard Centre for Integrative Neuroscience, University of Tübingen, Tübingen, Germany

**Keywords:** neuro-musculoskeletal model, motor control, optimality principles, hierarchical control, biomechanics, biorobotics, Bayesian optimization

## Abstract

Human arm movements are highly stereotypical under a large variety of experimental conditions. This is striking due to the high redundancy of the human musculoskeletal system, which in principle allows many possible trajectories toward a goal. Many researchers hypothesize that through evolution, learning, and adaption, the human system has developed optimal control strategies to select between these possibilities. Various optimality principles were proposed in the literature that reproduce human-like trajectories in certain conditions. However, these studies often focus on a single cost function and use simple torque-driven models of motion generation, which are not consistent with human muscle-actuated motion. The underlying structure of our human system, with the use of muscle dynamics in interaction with the control principles, might have a significant influence on what optimality principles best model human motion. To investigate this hypothesis, we consider a point-to-manifold reaching task that leaves the target underdetermined. Given hypothesized motion objectives, the control input is generated using Bayesian optimization, which is a machine learning based method that trades-off exploitation and exploration. Using numerical simulations with Hill-type muscles, we show that a combination of optimality principles best predicts human point-to-manifold reaching when accounting for the muscle dynamics.

## 1. Introduction

Goal-directed arm movement has been studied extensively in neuroscience with the aim of deriving a predictive model of human and animal movements (e.g., Bizzi et al., 1984; Flash and Hogan, 1985; Harris and Wolpert, 1998; Campos and Calado, 2009). It is widely accepted that the central nervous system (CNS) selects a specific movement to follow an optimal path, which minimizes certain costs to achieve the movement goal (Todorov and Jordan, 2002; Franklin and Wolpert, 2011). Still, it is unclear which criterion of optimality is chosen by the CNS while generating and controlling the motion. For point-to-point reaching tasks, several different isolated optimality criteria have been proposed, such as e.g., minimum hand jerk (Flash and Hogan, 1985), minimum torque change (Uno et al., 1989), minimum energy (Alexander, 1997), and minimum variance (Harris and Wolpert, 1998). In a more recent work, Berret et al. (2011b) used kinematic input data and reconstructed the optimality function for point-to-manifold movements in humans. Such point-to-manifold movements are interesting, as they allow for a richer set of solutions as compared to point-to-point movements (de Rugy et al., 2012; Kistemaker et al., 2014; Mehrabi et al., 2017). Berret et al. (2011b) found that only a combined cost function minimizing mechanical energy consumption and movement jerk (maximizing smoothness) allows to reasonably predict the trajectories of point-to-manifold movements.

In the study of Berret et al. (2011b), muscle forces acting on the respective joints are lumped to one joint torque per each joint. While this assumption is supported by the idea that muscles are grouped together to produce joint torque forming synergies of muscles (e.g., d'Avella et al., 2003), it neglects the contribution of the individual muscle to joint torque generation. Similar, in a very recent study by Oguz et al. (2018), free-space reaching motions were investigated by using joint torques representing muscle contractions. Both studies do not take into account the interaction of the individual, non-linear muscle dynamics with the non-linear dynamics of the skeleton. However, it is known that muscles with their characteristic activation dynamics, non-linearities, elasticities, and antagonistic setup contribute to the characteristics of biological movement (van Soest and Bobbert, 1993; Daley et al., 2009; Schmitt et al., 2019) which has consequences for the interpretation of the underlying motor control principles (Pinter et al., 2012). Thus, the question is whether individual muscle dynamics play a significant role in the optimality of motion generation and control for point-to-manifold tasks? More precisely, in comparison with Berret et al. (2011b) the question is, whether or not the composite optimality function found, still holds true, if muscle dynamics are considered, explicitly?

In this contribution, a neuro-musculoskeletal arm model (Bayer et al., 2017; Driess et al., 2018; Stollenmaier et al., 2020) is used to simulate arm movements. Point-to-manifold experiments are investigated numerically. The underlying control policy to generate arm movements is synthesized using different isolated, well-known optimality principles and combinations thereof. Due to the complexity of the movement apparatus, the optimality of a given control policy can only be evaluated by performing a simulation. Therefore, we propose to use Bayesian optimization as a sample efficient technique to optimize the cost function corresponding to a chosen optimality principle. Bayesian optimization uses a probabilistic surrogate model of the cost function to automatically trade-off exploitation and exploration according to a utility function. Thus, it can be interpreted as a form of reinforcement learning similar to the natural process in animal learning.

The purpose of this study is to investigate whether previously proposed cost functions allow to reproduce experimental data of human point-to-manifold movements. The novelty of our work is the use of a neuro-musculoskeletal model to synthesize optimal movement considering both isolated and combined cost functions and investigate the contribution of individual muscle dynamics in point-to-manifold movements.

## 2. Methods

Different optimality principles are applied to a two-joint biophysical arm model with six muscles, represented by Hill-type muscles (Günther et al., 2007; Haeufle et al., 2014), to investigate free endpoint movements. A point-to-manifold scenario is set up to distinguish between various cost functions. The arm movement is generated by finding a static, open-loop muscle stimulation set for all included muscle, using the selected optimality principle, to reach the manifold from a given, fixed starting point. Thus, let ξ be a trajectory of features (e.g., joint positions, velocities, torques, etc.) that is obtained by simulating an arm movement as a function of the static muscle stimulation *u*. The trajectory evolves solely from the dynamics of the musculoskeletal system. The optimization problem for the specified cost function *J* reads as

(1) $$\begin{array}{l} \underset{u\in U}{\min}J\left(\xi \left(u\right)\right) \end{array}$$

where $U={\left[0,1\right]}^{n}$ denotes the space of *n* possible muscle stimulations (in our case *n* = 6). Testing a new muscle stimulation involves the computationally expensive simulation of the arm system since no closed-form expression for ξ(*u*) exists. To address this challenge, we propose to find muscle stimulations in a sample efficient way via Bayesian optimization.

In the following, the single components of the workflow, namely the neuro-musculoskeletal arm model, the formulation of the optimality principles as cost functions, and Bayesian optimization are described. Furthermore, the general setup is shown.

### 2.1. Setup

**Figure 1:**
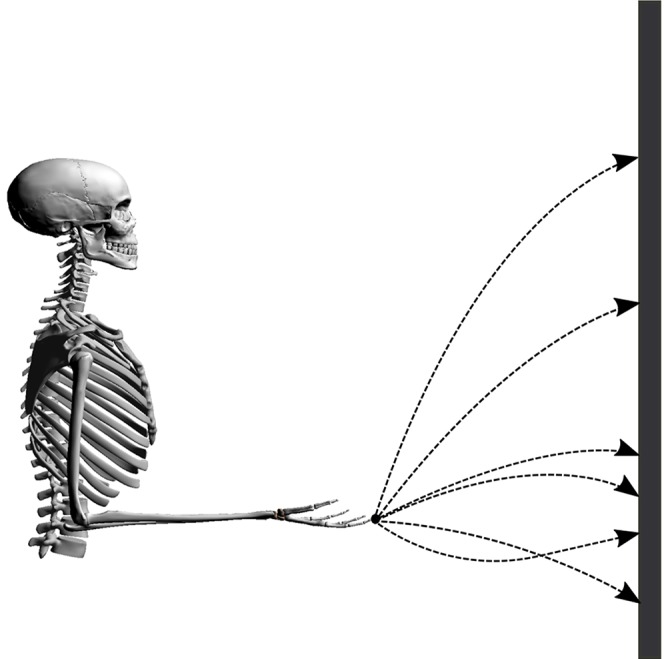
Illustration of the setup. Possible trajectories of the finger tip from the start position to the bar are shown in dashed lines.

**Figure 2:**
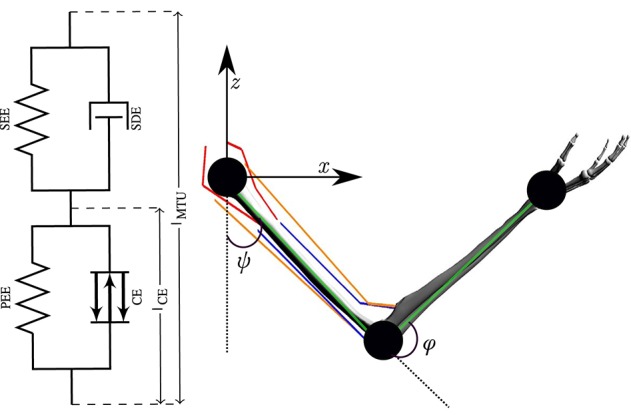
The numerical model of a human arm. The six muscles are modeled as lumped Hill-type muscles depicted on the left (figure adapted from Haeufle et al., 2014). On the right, the kinematic chain (green lines) with the two joints and the joint angles ψ and φ is shown. Red lines depict the two monoarticular shoulder muscles (ante- and retroversion), orange lines the two biarticular ones and blue lines represent the two monoarticular elbow muscles (flexor and extensor).

Point-to-manifold experiments are more suitable to distinguish between different cost functions than point-to-point experiments, as shown by Berret et al. (2011b). To validate the predictions of our model, we resort to previously published experimental data from Berret et al. (2011a). In this study, subjects were asked to point with a one-shot movement to a bar placed in front of them with closed eyes. In contrast to typical point-to-point experiments, the endpoint on the bar was not defined a priori but is freely chosen by the subjects. The numerical setup is established accordingly by placing the neuro-musculoskeletal arm model in front of a vertical bar, as visualized in Figure 1. The bar represents the target manifold in front of the subject at a distance of 85% of the total arm length (*L* = *l*_1_ + *l*_2_, where *l*_1_ and *l*_2_ denote upper arm and forearm lengths, respectively). Every simulation starts from the same given set point with zero initial velocities and an arm posture of φ = 90° for the elbow and ψ = 0° for the shoulder angle. This initial condition can be seen in Figure 1, the angles are defined in Figure 2. These values are chosen to mimic the experimental setup from Berret et al. (2011b). The initial condition for the muscles was chosen to minimize the sum of muscle stimulation to resemble a relaxed starting position (Bayer et al., 2017). Applying an open-loop stimulation u∈U then results in the execution of a dynamic movement. The trajectory and endpoint equilibrium position depend on the chosen stimulation *u*.

#### 2.1.1. Point-to-Manifold

We define the point-to-manifold scenario for our study as follows:

(2) $$\begin{array}{ll} x\left(0\right)=x_{0}, & \;ẋ\left(0\right)=0,\\ z\left(0\right)=z_{0}, & \;ż\left(0\right)=0,\\ x\left(T\right)=x^{⋆}, & \;ẋ\left(T\right)=0,\\ z\left(T\right):\text{arbitrary} & \;ż\left(T\right)=0. \end{array}$$

Here, *x* and *z* are the hand positions in the respective directions for the starting time *t* = 0 and the movement duration *t* = *T*, ẋ and ż denote the time derivatives of these quantities. Furthermore, *x*^⋆^ stands for the desired horizontal end position. Note, that in contrast to point-to-point movements, here the desired *z* position is a random goal point within the manifold spanned by the *z* axis.

### 2.2. Musculoskeletal Model

The numerical arm model consists of two segments representing the upper and lower arm, which are driven by six muscles, two monoarticular muscles each for the shoulder and the elbow joint, as well as two biarticular muscles acting on both joints (Driess et al., 2018, see Supplementary Material for more details). The parameters are based on previous publications (Kistemaker et al., 2007; Bayer et al., 2017). The upper body is fixed in space, and a hinge joint connects the two segments. The limitation to planar movements is justified, as it has been shown in the analysis of experimental data that the movements mostly lay along the para-sagittal plane (Berret et al., 2011a). The dynamics of the skeletal system are modeled as rigid bodies solving the Euler-Lagrange equation

(3) $$\begin{array}{l} M\left(\theta \right)\ddot{\theta }+C\left(\theta ,\overset{\cdot }{\theta }\right)=F\left(\theta ,\overset{\cdot }{\theta },t\right), \end{array}$$

where *M*(θ) is the mass matrix, θ = [φ, ψ] contains the elbow and shoulder angle, respectively, $C\left(\theta ,\overset{\cdot }{\theta }\right)$ consists of the centrifugal, gravitational and Coriolis forces and *F* denotes all components of the muscle-tendon forces acting on the arm. Muscle forces acting on the segments are predicted by Hill-type muscle models (Haeufle et al., 2014). This means that the muscle-tendon unit (MTU) is modeled with spring-damper elements consisting of four components (Figure 2): a contractile element (CE) modeling the force-length and force-velocity properties of active muscle fibers, a parallel elastic element (PEE), a serial elastic element (SEE), and a serial damping element (SDE). The underlying non-linear dynamics of the muscle model can be formulated as follows

(4a) $$\begin{array}{l} {\overset{\cdot }{l}}_{\text{CE}}=f_{v}\left(l_{\text{CE}},l_{\text{MTU}},{\overset{\cdot }{l}}_{\text{MTU}},a\right) \end{array}$$

(4b) $$\begin{array}{l} F_{\text{MTU}}=f_{f}\left(l_{\text{MTU}},{\overset{\cdot }{l}}_{\text{MTU}},a,l_{\text{CE}},{\overset{\cdot }{l}}_{\text{CE}}\right). \end{array}$$

Here, the first-order differential equation describes the contraction dynamics of the contractile element ${\overset{\cdot }{l}}_{\text{CE}}$, which is integrated in the calculation of the force of the muscle-tendon unit *F*_MTU_. The muscle's force depends on the current contraction state of the muscle *l*_CE_, the length of the muscle-tendon unit *l*_MTU_, and the muscle activity *a*. The relation between a neural stimulation signal *u* and the muscle activity *a* is a complex biochemical process which is approximated here by Hatze's model of activation dynamics (Hatze, 1977). Thus, the muscle activity *a*, which represents the free calcium ion concentration in the muscle, can be predicted with a first-order differential equation

(5) $$\begin{array}{l} ȧ=f_{a}\left(a,l_{\text{CE}},u\right). \end{array}$$

To generate the stimulation signal *u* ∈ [0, 1]^6^, an open-loop controller is implemented, which ensures that the simulated arm movements always terminate in a static equilibrium with a vanishing net joint moment (Bayer et al., 2017). The stimulations *u* are selected based on the chosen optimality principle with Bayesian optimization.

Performing a forward dynamic simulation with this arm model results in a feature matrix ξ(*u*)

(6) $$\begin{array}{l} \xi \left(u\right)={\left(\theta_{i}\left(t\right),\overset{\cdot }{\theta_{i}}\left(t\right),\ddot{\theta_{i}}\left(t\right),\overset{.}{\theta_{i}}\left(t\right),x\left(t\right),z\left(t\right),\overset{.}{x}\left(t\right),\overset{.}{z}\left(t\right),\tau_{i}\left(t\right),{\overset{\cdot }{\tau }}_{i}\left(t\right),u\right)}_{t=0}^{T}\\ \text{ for }i=1,2. \end{array}$$

The single components of ξ(*u*) are trajectories in time *t* and represent different physical quantities, such as the joint angles θ = [φ, ψ], the hand position in *x*- and *z*-direction, the torques τ_1_ and τ_2_ (acting on the two joints, elbow and shoulder, respectively) and time derivatives of these quantities. Note that all the results are shown for a non-fixed movement duration *T* (if not otherwise mentioned). This is due to the fact that open-loop muscle stimulations were found, which ensured that a steady state is always reached at the end of the arm movement. Therefore, the simulation was set up such that the model simulates until the arm velocity drops below a threshold value (10^−4^ m/s) and then terminates because the equilibrium state is reached.

### 2.3. Optimality Principles

Several cost functions have been proposed in the literature to investigate human arm movement with optimality principles. The most common ones are presented and compared here. Based on the evaluated state variables (i.e., components of feature matrix ξ), they are divided into five general groups. First, we consider *kinematic models*, e.g., the minimum-jerk model in joint and Cartesian-space coordinates (Flash and Hogan, 1985; Wada et al., 2001) and the minimum angle acceleration model (Ben-Itzhak and Karniel, 2008). They penalize high-order derivatives which in turn maximize the smoothness as introduced by Todorov and Jordan (1998). Historically, the minimum-jerk model was one of the most influential theories in motor control theory which was able to reproduce many of the experimental observations in real-human movements. However, kinematic models do not take anatomical constraints or non-linear arm characteristics into account. Therefore, *dynamic models* were proposed. In the literature, two cost variables are formulated at the dynamic level, namely the minimum torque (Nelson, 1983) and the minimum torque change model (Uno et al., 1989; Nakano et al., 1999). Although it might not seem intuitively important to optimize the torque change in biological systems, it was argued that the minimization of wear and tear on the musculoskeletal system is desired. On the contrary, the necessity of energy efficiency in the biological system is evident. Therefore, *energetic models* were proposed. One approach could be to minimize the metabolic energy consumed by the muscles, which is not considered here. Instead, the total absolute work was formulated as a cost function which is related to the mechanical energy (Berret et al., 2008). Alternatively, a more robotic approach, such as minimizing the control effort, can be used. Typically, using *control effort models* helps to handle redundancies. In this case, the amount of motor neuron activity is optimized by penalizing the sum of the squared muscle activations (Guigon et al., 2007). Furthermore, the class of *hybrid models* combines several single optimality principles. This work specifically focuses on the hybrid cost function proposed by Berret et al. (2011a) and Hilt et al. (2016). This model combines an energy term with a smoothness expression (e.g., angle jerk) and is able to predict free-endpoint arm movements. Our hypothesis was that due to the use of muscle dynamics, an additional term for the hybrid cost function might be necessary. We propose to include the control effort term (see *J*_JEE_ in Table 1) as it is the only single cost function term that directly affects muscle dynamics by taking the muscle stimulations into account. An overview of the cost functions used in this study is given in Table 1.

**Table 1:** Cost functions as proposed in literature.

**Optimality principle**	**Mathematical description**
Angle acceleration (Ben-Itzhak and Karniel, 2008)	$J_{\text{ACC}}=\int_{0}^{T}\left({\ddot{\varphi }}^{2}+{\ddot{\psi }}^{2}\right)\;dt$
Hand jerk (Flash and Hogan, 1985)	$J_{\text{HJ}}=\int_{0}^{T}\left({\dddot{x}}^{2}+{\dddot{z}}^{2}\right)\;dt$
Angle jerk (Wada et al., 2001)	$J_{\text{AJ}}=\int_{0}^{T}\left({\dddot{\varphi }}^{2}+{\dddot{\psi }}^{2}\right)\;dt$
Torque (Nelson, 1983)	$J_{\text{T}}=\int_{0}^{T}\left(\tau_{1}^{2}+\tau_{2}^{2}\right)\;dt$
Torque change (Uno et al., 1989; Nakano et al., 1999)	$J_{\text{TC}}=\int_{0}^{T}\left(\dot{{\tau_{1}}^{2}}+\dot{{\tau_{2}}^{2}}\right)\;dt$
Energy (Berret et al., 2008)	$J_{\text{EN}}=\int_{0}^{T}\left(\left|\dot{\varphi }\cdot \tau_{1}|+|\dot{\psi }\cdot \tau_{2}\right|\right)\;dt$
Effort (Guigon et al., 2007)	$J_{\text{EFF}}=\sum_{i=1}^{6}u_{i}^{2}$
Hybrid jerk and energy (Berret et al., 2011a; Hilt et al., 2016)	$J_{\text{JE}}=\int_{0}^{T}\left(\left|\dot{\varphi }\cdot \tau_{1}|+|\dot{\psi }\cdot \tau_{2}\right|\right)\;dt+10^{-3}\cdot \int_{0}^{T}\left({\dddot{\varphi }}^{2}+{\dddot{\psi }}^{2}\right)\;dt$
Hybrid jerk, energy, and effort	$J_{\text{JEE}}=\int_{0}^{T}\left(\left|\dot{\varphi }\cdot \tau_{1}|+|\dot{\psi }\cdot \tau_{2}\right|\right)\;dt+10^{-3}\cdot \int_{0}^{T}\left({\dddot{\varphi }}^{2}+{\dddot{\psi }}^{2}\right)\;dt+\sum_{i=1}^{6}u_{i}^{2}$

#### 2.3.1. External Task Constraint

To ensure that the task constraints of pointing to a vertical bar are fulfilled, the desired end position is imposed. This is done by extending the cost function with an additional term. The total cost function is then defined as

(7) $$\begin{array}{l} J_{\text{total}}=\|x_{T}-x^{⋆}{\|}^{2}+0.01\cdot J_{\text{opt}} \end{array}$$

where *x*_*T*_ denotes the reached *x*-position of the hand in equilibrium and *x*^⋆^ stands for the desired horizontal end position (location of the bar). Note that the relation between the task constraint and the chosen optimality principle has the same magnitude as suggested by Li and Todorov (2007).

### 2.4. Finding Muscle Stimulations via Bayesian Optimization

As discussed in section 2, the goal is to find static muscle stimulations $u\in U\subset {\mathbb{R}}^{6}$ that, when applied to the neuro-musculoskeletal system, minimize the specified cost function *J*. However, no analytical form of *J*, i.e., no gradient in particular, is known, instead, *J* can only be queried for specific choices of *u*, which involves the computationally expensive forward dynamic simulation of the system, cf. section 2.2. Therefore, the optimization procedure is an episodic process that seeks for an optimal set of muscle stimulations based on the information gathered so far. In this way, there are parallels between the situation in the present work and real-world motor learning tasks, where humans improve their skills by trial and error (Taube et al., 2008).

Bayesian optimization (Brochu et al., 2010) addresses these problems in a sample efficient manner, by learning a probabilistic surrogate model of the cost function *u* ↦ *J*(ξ(*u*)) based on collected data $D_{n}={\left\{\left(u_{i},J\left(\xi \left(u_{i}\right)\right)\right)\right\}}_{i=1}^{n}$ obtained from *n* previous episodes. (The cost function model can be interpreted as an internal model of a biological system.)

A common choice for the probabilistic surrogate model are so-called Gaussian processes (Rasmussen and Williams, 2004), which describe the probability density of *J*(ξ(*u*)) given the current dataset $D_{n}$ as a Gaussian distribution

(8) $$\begin{array}{l} P\left(J\left(\xi \left(u\right)\right)|D_{n}\right)=N\left(J\left(\xi \left(u\right)\right)|\mu_{n}\left(u\right),\sigma_{n}^{2}\left(u\right)\right) \end{array}$$

with mean $\mu_{n}\left(u\right)=\kappa {\left(u\right)}^{T}(K_{n}+\varepsilon^{2}I_{n}{)}^{-1}y_{n}$ and variance $\sigma_{n}^{2}\left(u\right)=k\left(u,u\right)-\kappa_{n}{\left(u\right)}^{T}(K_{n}+\varepsilon^{2}I_{n}{)}^{-1}\kappa_{n}\left(u\right)$, where $\kappa_{n}\left(u\right)={\left(k\left(u,u_{i}\right)\right)}_{i=1}^{n}$, $K_{n}={\left(k\left(u_{i},u_{j}\right)\right)}_{i,j=1}^{n}$, $y_{n}={\left(J\left(\xi \left(u_{i}\right)\right)\right)}_{i=1}^{n}$. In this work, we use the common squared exponential kernel k:U×U→ℝ with $k\left(u,u^{\prime }\right)=\alpha \exp\left(-\gamma \|u-u^{\prime }{\|}_{2}^{2}\right)$. The choice of the kernel and its hyperparameters encodes the correlation between data points and thereby the complexity/smoothness of the surrogate model. In this case, the hyperparameters are the length scale γ ∈ ℝ and signal variance α ∈ ℝ.

Based on the information encoded in the Gaussian process model, Bayesian optimization selects the next query point *u*_*n*+1_ for the next episode by maximizing an acquisition function *a*

(9) $$\begin{array}{l} u_{n+1}=\underset{u\in U}{\mathrm{argmax}}a\left(u;D_{n}\right). \end{array}$$

In the vicinity of the already collected stimulations, the model has high certainty, reflected in a low variance $\sigma_{n}^{2}\left(u_{n+1}\right)$. This knowledge can be exploited by querying the cost function at a point of high certainty and low predicted cost. However, there might be unexplored regions in U with low costs that the current model is unaware of, i.e., has high uncertainty. This trade-off between exploring U and minimizing *J* based on the current information in the probabilistic model is formalized in the upper confident bound acquisition function

(10) $$\begin{array}{l} a_{\text{UCB}}\left(u;D_{n}\right)=\beta \sigma_{n}\left(u\right)-\mu_{n}\left(u\right), \end{array}$$

where β ∈ ℝ controls this exploration/exploitation tradeoff.

In all experiments, the tradeoff parameter was β = 0.01, the kernel hyperparameters α, *l* were optimized with L-BFGS by maximizing the data likelihood. The dataset was initialized with 10 random muscle stimulations sampled uniformly in U. The optimization of the acquisition function was also performed with L-BFGS using 30 random restarts, again uniformly sampled in U. The algorithm terminates after a fixed number of iterations (maxIter), in this case, after 600 iterations, which seems to be a good choice for this problem setting, as shown in section 3.3.

The pseudo-code of this algorithm is shown in Table 2. Bayesian optimization has empirically been shown to be a sample efficient method for optimizing black-box cost functions, e.g., in real world robotic applications (Marco et al., 2016; Drieß et al., 2017).

**Table 2:** Bayesian optimization algorithm.

**Algorithm**
initialize data set $D_{0}$ with 10 random samples
**for** n = 1,2,…,maxIter **do**
select muscle stimulation $u_{n}\in {\mathbb{R}}^{6}$ by optimizing the acquisition function *a*_UCB_
$u_{n}=\underset{u\in U}{\arg\max}a_{\text{UCB}}\left(u;D_{n-1}\right)$
Run dynamic simulation of musculoskeletal system to obtain ξ(*u*_*n*_)
Evaluate the cost function *J*(ξ(*u*_*n*_))
Augment the data $D_{n}=D_{n-1}\cup \left\{\left(u_{n},J\left(\xi \left(u_{n}\right)\right)\right)\right\}$
Update Gaussian process model of the cost function
**end for**

## 3. Results

### 3.1. Predicted Trajectories

**Figure 3:**
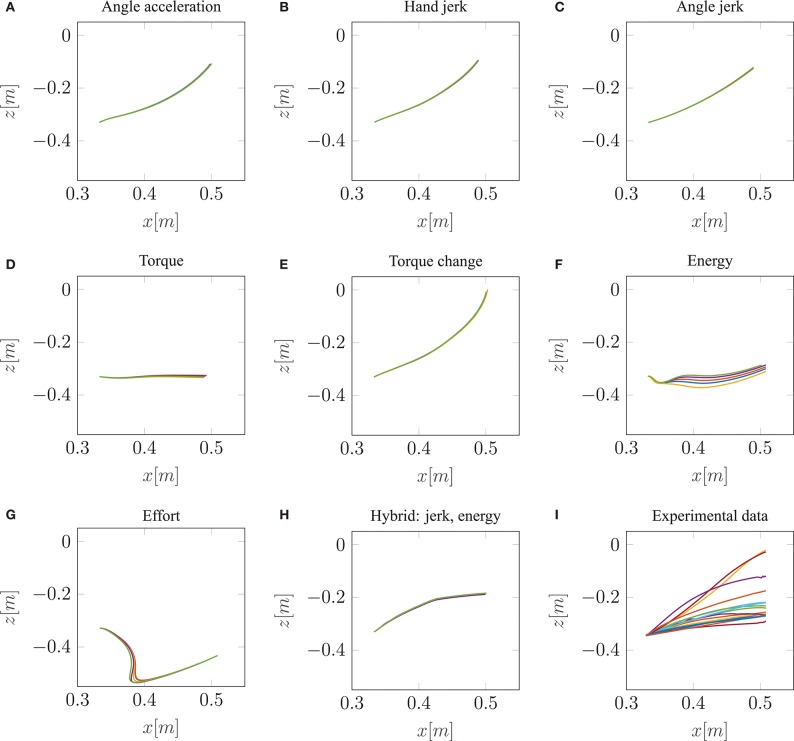
The best five predicted trajectories for the fingertip movement ending on the bar are shown. Eight different cost functions are compared with the experimental trajectories, in analogy to the metric of Berret et al. (2011a), which is based on Cartesian (endpoint of trajectory) and curvature errors (see section 3.1).

Our neuro-musculoskeletal model predicts eight different trajectories, one for each optimality principle. The first eight subplots in Figures 3A–H show the best five simulated trajectories corresponding to the five best *u* of each cost function, which were found using Bayesian optimization. The last plot on the lower right in Figure 3I, shows the recorded experimental data for 17 subjects as collected by Berret et al. (2011a). Note, that the data was post-processed in the same way as in the paper from Berret et al. (2011a): The signals were low-pass filtered using a digital fifth-order Butterworth filter at a cutoff frequency of 10 Hz. Furthermore, the on- and offset of the movement were defined at the time points where the linear tangential velocity of the fingertip exceeded 5% of its peak velocity, and respectively dropped below. The graphs show that the predicted finger paths differ for the different optimality principles (subplots Figures 3A–H). This is not surprising as, in contrast to typical point-to-point tasks, the point-to-manifold experiment allows more freedom. Another point to be mentioned is the similarity between the angle acceleration model (Figure 3A), the hand jerk model (Figure 3B), the angle jerk model (Figure 3C), and the torque change model (Figure 3E). This behavior can be explained by the fact that all four models maximize the smoothness of movements. Figure 3D displays the results using the torque model. Based on this optimality principle, the arm points more or less on a straight path toward the bar and predicts a much lower endpoint on the bar compared to the experimental data. Similar to the torque model, the energy model predicts a lower endpoint on the bar (Figure 3F). Furthermore, the general curvature is different to the experimental data. Instead of having a concave trajectory as shown in the experimental data, the energy model predicts trajectories which first drop downwards, before pointing forward. It is also interesting to observe the effort model (Figure 3G) for which the simulated trajectory first falls strongly and then points upwards to the bar. Therefore, this model is the only one which predicts a lower endpoint on the bar than the original start point. As prioritized by the cost function, this model uses the lowest muscle activations to control the movement in comparison to all other model predictions. However, none of the optimality principles with a single cost term reproduces the experimental trajectories as well as the hybrid model *J*_JE_.

Similar to the results of Berret et al. (2011a), our model can predict biological behavior more realistically with the hybrid model (Figure 3H) in comparison to all single-cost optimality principles. For the hybrid model, the endpoint, as well as the general curvature, match the experimental data well (c.f. Figure 3I). For the comparison between the simulated and the experimental trajectories, it is still an open question in motor control how to define a metric that includes all important movement features (Gielen, 2009). One metric, which was proposed by Berret et al. (2011a), is a sum of measuring the Cartesian and curvature errors between the simulated and the experimental trajectories. They discussed that based on human intuition, it is important to include both the shape of the path and the endpoint position. Due to this metric, we analyzed all the endpoints and curvatures of the simulated trajectories visually, as shown in Figure 3. Furthermore, we performed a quantitative analysis, where we computed the endpoint error on the bar and the maximum signed curvature error as a measure of convexity or concavity of a trajectory. The results of this quantitative analysis are shown in the Table S1 and Figure S2. To summarize this analysis, looking at both trajectory metrics, the hybrid jerk and energy model has the lowest error compared to the experimental data for all cost functions presented in Figure 3.

To conclude, the results presented above show the behavior of different single cost functions. None of them is able to match both the curvature and the endpoint of the experimental data well. The predicted trajectory of the hybrid jerk and energy model is the closest to real human behavior w.r.t. the endpoint error and curvature error, which is the reason why this cost function is investigated in more detail in the following.

### 3.2. Influence of Muscle Stimulations on Tangential Velocities

**Figure 4:**
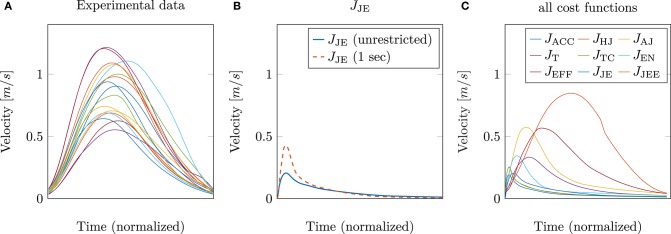
**(A)** The experimentally measured tangential velocities of 17 subjects are compared to the simulated velocities using **(B)** the hybrid *J*_JE_ model and **(C)** all cost functions including the extended hybrid *J*_JEE_ model (1 s) for the best prediction.

So far, only the position trajectory has been analyzed and discussed. The next step is to investigate whether the hybrid model (jerk and energy) is also able to predict other kinematic features, such as the tangential velocity correctly. This is shown in Figure 4. On the left, the experimental tangential velocities (again 17 subjects) are shown in comparison to the velocity curves of the model with the best trajectory prediction, i.e., the hybrid model *J*_JE_ (solid blue line in Figure 4B). It is striking that both the peak as well as the general curvature, are significantly different. This is contrary to the results of Berret et al. (2011a), where the hybrid model was able to match the experimental velocities well. An explanation for these differences could be that in our study, muscle stimulations are used as control variables instead of controlling the torques directly. Another point is that the movement duration was not restricted. Previous investigations (e.g., Gribble and Ostry, 2000; Kistemaker et al., 2006; Shadmehr, 2010; Berret et al., 2011a; Pinter et al., 2012) usually fixed the movement duration. To show how this affects the results, we additionally implemented a limitation of the movement duration to 1 s, which corresponds to the experimental movement duration. This was done by terminating the simulation after 1 s. To ensure that the velocity at the endpoint is still zero, an additional term was added to the external task constraint in *J*_total_. Restricting the movement duration also takes into account that slow movements are favored by the jerk model due to the fact that the jerk cost approaches zero for an infinite movement duration. However, this restriction still leads to a right-skewness in the curvature of the predicted tangential velocities, as shown in Figure 4B (dashed red line). Consequently, the difference in modeling the arm by including muscle dynamics was taken into consideration. As mentioned previously (section 2.2), the Hatze activation function was used for modeling the activation dynamics of the muscles. This function has the property that high muscle stimulations only need a short time to reach peak activity, while the time to decrease is longer (Rockenfeller et al., 2015; Bayer et al., 2017). Indeed, some of the chosen muscle activations based on the hybrid model are very high, e.g., the monoarticular anteversion shoulder muscle (MSA) is activated with *u* = 1. This explains the strong asymmetrical behavior of the tangential velocity (Figure 4B).

**Figure 5:**
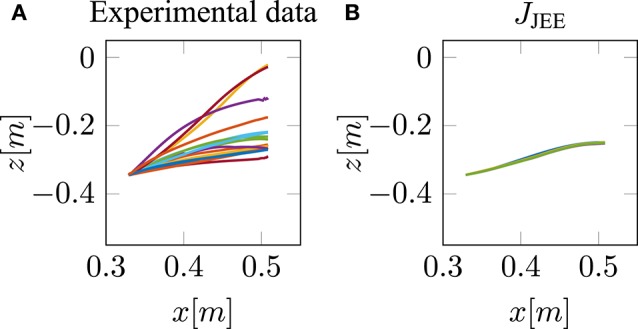
Comparison between the experimental trajectories and the new cost function *J*_JEE_.

This is in line with our hypothesis, as mentioned above in section 2.3 that it is necessary to restrict the search space by selecting low activated muscle stimulations. Therefore, we proposed to add an effort term to the hybrid cost function, which favors a small sum of squared muscle stimulations (*J*_JEE_, last row of Table 1). This additional term directly affects and takes the muscle dynamics into account. As shown with the *J*_JEE_ line (orange) in Figure 4C, this leads to movements with a realistic bell-shaped velocity curve with a peak velocity of 0.85*m*/*s*. This is comparable to experimental data. All other tangential velocities shown in Figure 4C have smaller peak velocities and show more right-skewness in their velocity profiles compared to both the experimental data and the *J*_JEE_ function. Furthermore, Figure 5 shows that the predicted finger path of the new cost function *J*_JEE_ is similar to the experimental results regarding two significant movement features: the Cartesian error (endpoint of the trajectory) and the general curvature error (based on the metric of Berret et al., 2011a). Taken these two movement features together with the movement features of the velocity curve (see Figure 4C: bell-shaped profile and peak velocity matches), this supports our hypothesis that the additional effort term should be included in the cost function *J*_JEE_. In addition, we performed a quantitative analysis for the two trajectory movement criteria (as mentioned above) and for two velocity movement criteria for all cost function including the final proposed cost function *J*_JEE_. The quantitative analysis of the velocity profiles consists of the peak velocity error and the skewness error (measuring bell-shapedness or left- or right-skewness) in comparison to the experimental data. The results are shown in Table S1 and Figure S2. For all movement criteria, the *J*_JEE_ cost function has either the lowest or a very small error compared to the other presented models.

Summed up, the results show that our model can predict biological behavior more realistically if the muscle activation is taken into account.

### 3.3. Performance of Bayesian Optimization

**Figure 6:**
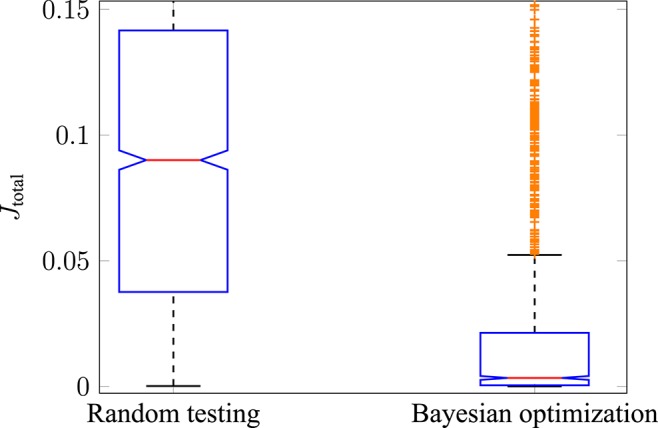
Performance of Bayesian optimization. The total error using random testing (left boxplot) is compared to using Bayesian optimization (right boxplot). The median of all observations is shown with a central red line, and the blue boxes represent the 25th to 75th percentile. The tested muscle activations sampled by Bayesian optimization result in a significantly lower error compared to randomly drawn stimulations.

The performance of Bayesian optimization in comparison to random testing was investigated. The reason for this is to show that the optimization is better than simply randomly sampling the search space of $u\in U\subset {\mathbb{R}}^{6}$. The results for all cost functions were similar, therefore, they are shown using the example of the hybrid model (*J*_JE_). To do so, three test runs were performed using random testing (each run with 600 iterations) and then compared to three test runs using Bayesian optimization (each run with 600 iterations). The resulting cost function *J*_total_ for both cases is shown with boxplots in Figure 6. It can be stated that the median of *J*_total_, indicated by the red central line, for random testing (left side) is significantly higher compared to using Bayesian optimization (right side). Furthermore, the 25th to 75th percentiles, also called the interquartile ranges (IQR), are in different magnitudes as indicated by the blue boxes. In this case, the maximum whisker length *w* is 1.5 times the IQR. This means that points are classified as outliers if they are greater than *q*3 + *w*·(*q*3 − *q*1) or less than *q*1 − *w*·(*q*3 − *q*1), where *q*1 and *q*3 are the 25th and 75th percentiles of all drawn observations. It is interesting to note that most points classified as outliers in the Bayesian optimization case (shown as orange crosses) are still part of the interquartile range in the case of random testing. Additionally, it can be shown that the mean values of the two test scenarios are from different populations by using a statistical hypothesis test with the Student's t-distribution. The *H*_0_-hypothesis that the two test runs have an equal mean value is rejected with a significance level of α = 0.01. Therefore, it can be stated that Bayesian optimization is better than random testing for sampling muscle activations under the consideration of different optimality principles.

**Figure 7:**
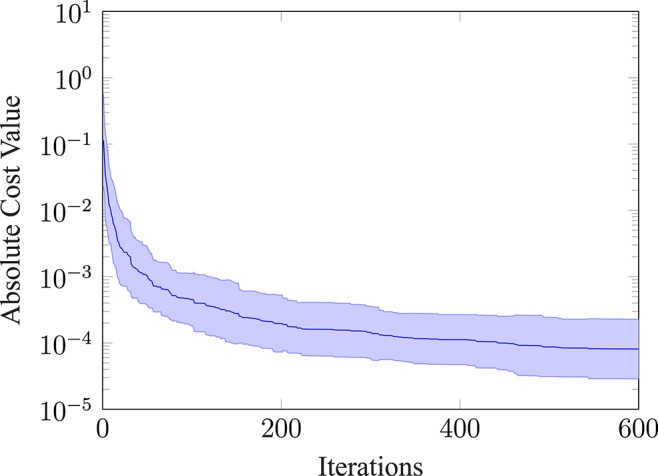
Absolute cost value of the best observation. Plotting the mean and the standard deviation (shaded area) for 30 repeated runs.

Furthermore, we evaluated how the absolute cost value of the best evaluation changes over the iterations for thirty repeated runs. The mean and the standard deviation of this evaluation is shown in Figure 7. The absolute cost value drops at the beginning and then settles on a mean value of around 8.09e − 5. Note, that we would not expect the absolute cost value to go to zero, because the movement has a cost and rather converges toward a finite value. Furthermore, the absolute value of the standard deviation (shown as the shaded area) narrows down, the more iterations are performed.

## 4. Discussion

In this study, we hypothesized that a combination of optimality principles determines human point-to-manifold reaching and that the muscle dynamics have an influence on the investigation of optimality. For this purpose, we applied several cost functions to a forward dynamics simulation of a muscle-driven arm model. The cost functions are minimized using Bayesian optimization, which searches for optimal open-loop muscle stimulations. We showed that a mixed cost function minimizing mechanical work, jerk, and neuronal stimulation effort simultaneously can replicate the participants' behavior in this task much better than any other of the investigated single cost criteria (Figure 3).

In the human arm, all sources of mechanical energy to drive the movement lie in the muscle-tendon unit (MTU). All actions of the MTU are triggered by motor commands of the central nervous system (CNS) sent directly to the individual muscle fiber within the MTU over neural pathways. Additionally, the MTU sends sensory signals back to the CNS. Thus, the MTU is the crucial link between the neuronal communication of the CNS and the physical interaction within the body's structure and the environment. In literature, several authors highlight the contribution of muscle properties to the control of motion, e. g., in jumping (van Soest and Bobbert, 1993), hopping (Haeufle et al., 2010), animal running (Daley et al., 2009). For studying neuroscience, however, it is still unclear which features to include into a mathematical model of a biological motion system. Pinter et al. (2012) compared arm models with actuators of different levels of detail – from a plain torque generator to a model actuated by four macroscopic Hill-type MTUs. They demonstrated that the response to perturbations varies and conclusions on control concepts may be inadequate if the macroscopic muscle characteristics are not considered. The findings of this work are in line with the literature. By using an arm model including individual muscles and, at least, a macroscopic model formulation of the muscles' dynamics, the arm kinematics change, significantly. We are not the first to mention that the choice of the used biophysical model and its level of detail to study motion generation and control is sensible as mentioned above, however, we recommend to include explicit formulations of the muscles' dynamics (Kistemaker et al., 2014; Mehrabi et al., 2017). For example, the velocity profile of the arm kinematics changed dramatically (Figure 4), just by accounting for appropriate muscle stimulations in the cost function.

In combination with these Hill-type muscles, we used an open-loop control approach to investigate optimality principles. This means no trajectories were planned, nor did we perform an inverse dynamics calculation (internal inverse model). Furthermore, open-loop control, in this case, means no sequence of muscle activations was used because setting only one set of scalar muscle stimulations is sufficient to produce trajectories (Figures 3, 5). This is different from some of the previous investigations (e.g., Kawato et al., 1987; Wolpert et al., 1995; Todorov and Jordan, 2002; Berret et al., 2011a) where closed-loop control or inverse simulations were used to analyze different cost functions. We think the assumption that feedback does not play a large role in this experiment is justified (Shadmehr et al., 2010; Oguz et al., 2018) because the participants had closed eyes without any external perturbations. Furthermore, the lack of feedback corrections means that the controller also acts as a planner (internal forward model) because it predicts the arm motion for a selected control signal. Summed up, we showed that it is possible to generate trajectories and investigate optimality with a simple open-loop control (see Figure 3).

Another important aspect of the controller is not only investigating optimality but also fulfilling the task, in this case, point-to-manifold reaching. Point-to-manifold reaching allows discriminating between cost functions which is shown in Figure 3. This is important because other tasks, like the intensely studied point-to-point reaching task, may result in similar behavior for different cost functions resulting in the conclusion that cost functions may be interchangeable (Nelson, 1983; Kistemaker et al., 2014; Spiers et al., 2016). Tasks like point-to-manifold reaching with a more openly defined target have a higher potential for revealing differences in the optimality principles as they result in different trajectories. This was also discussed by Berret et al. (2011a) where they showed, as a proof of concept, that hand jerk and torque change cost functions are much more distinguishable in point-to-manifold than in point-to-point reaching. Furthermore, we performed point-to-point simulations with a similar setup described above from the point-to-manifold simulations (see Figure S1). As shown there, almost all criteria predict the two typical movement features for point-to-point reaching movements: straight paths and bell-shaped speed profiles similar to previous findings in the literature (e.g., Abend et al., 1982; Flash and Hogan, 1985; Harris and Wolpert, 1998; Todorov, 2004). This makes it almost impossible to decide which cost function is the true one based on the given task since they all have a good theoretical basis and predict very similar trajectories. Therefore, it can be stated that conclusions on optimality principles depend, at least partly, on the chosen task.

Using this openly defined task, we showed that a combination of smoothness, energy, and effort seems to be a good choice as optimality principle for selecting a trajectory (Figure 5). Many arguments have been made to give an understanding of why each of the single cost criteria gives an advantage to the survival of the fittest (for an overview see Todorov, 2004). It is often argued that while energy is a limited resource in our system, it is important to minimize its consumption (Hatze and Buys, 1977; Alexander, 1997; Berret et al., 2008), whereas smoothness can be interpreted as a measure of the prevention of self-injuries of the musculoskeletal system (Todorov and Jordan, 1998). A combination of these two principles was already proposed by Berret et al. (2011a). However, we found that by including muscle dynamics, the cost function needs to be adapted, as well. If muscle stimulation represents a physiological signal, like the muscle membrane potential in our case, we found that the interpretation of control effort is more plausible and physiologically valid. Therefore, including the cost of muscle stimulation into the cost function (*J*_JEE_, last row of Table 1) is not only necessary but allows for a more realistic search for the underlying optimality principles, as well. Additionally, such an enhanced cost function allows for an implicit integration of earlier findings regarding movement optimality, such as reduction of noise (Harris and Wolpert, 1998), because noise scales with the control signal. Furthermore, it was mentioned by McKay and Ting (2012) that similar muscle activity patterns are predicted by cost functions, such as reduction of signal-dependent noise compared to the minimization of control effort. This would further support our findings. Concluding, a combination of these cost functions is reasonable, and evidence for this combination is shown in this work (see Figure 5).

Considering this influence of the muscles on the selection of the optimality principle, the question arises if other implicit aspects also have an influence? In this study, we showed that transferring a real task to a valid simulation task also leaves some other parameters open to be set, such as movement duration (see Figure 4). It is unclear how the non-specific task requirement of pointing fast is translated into a quantitatively measured time. Some authors (e.g., Tanaka et al., 2006) argued that movement duration is minimized under the constraint that the endpoint accuracy of the movement is still good enough based on Fitts's law (Fitts, 1954). However, in this openly defined target we used in this work, the accuracy is not given explicitly, which in turn makes it difficult to set a movement end time. Therefore, we first choose an open subset of possible solutions by simulating the movement until an equilibrium endpoint is reached. However, we have seen that restricting the movement duration from an equilibrium endpoint to 1 s, consequently, also changed the tangential velocities. Setting this new end time which is closer to the experimental movement durations, affected the simulated tangential velocities such that they matched the experimental ones better (Figure 4). This shows that it is not clear how implicit task aspects, such as time are incorporated in the biological structure nor how they can be modeled.

Another point which is important for investigating muscle-actuated synthesized movement is that not only the initial angles or initial end-effector position determine the system state but rather the pre-activation of the muscles needs to be included as well. In another study by Bayer et al. (2017), it was shown that the pre-activation of the muscles has a strong effect on the maximum movement velocity. Therefore, we chose the minimum sum of muscle stimulations as the initial condition. This can be interpreted as a “relaxed” starting state. Taken this together with the previously discussed time aspect (Figure 4), we want to emphasize that through external factors or non-specific task requirements, the arm movement control is changed. In this context, by external factors, we mean both the environment as well as the given task. Here, the environment includes, e.g., external perturbations, joint limits, obstacle avoidance, and many more. Both the environment and the given task can influence the movement features, such as speed and movement duration, accuracy, distance and amplitude, noise and the initial condition. Connecting these points, this supports the hypothesis that optimality is a restricted function in the domain of task and environment.

## Data Availability Statement

The datasets generated for this study are available on request to the corresponding author.

## Author Contributions

IW, SS, and MT: project concept. IW: numerical experiments. IW and SS: analyzed the data. IW, DD, HZ, DH, and SS: contributed methods/code. IW, DD, HZ, DH, MT, and SS: wrote the paper.

## Conflict of Interest

The authors declare that the research was conducted in the absence of any commercial or financial relationships that could be construed as a potential conflict of interest.

## Supplementary Material

The Supplementary Material for this article describes the Arm26 model which was used in this study (Stollenmaier et al., 2020). Furthermore, it contains results for point-to-point simulations to show the advantage of using a more openly defined task, such as the point-to-manifold task. Additionally, we performed a quantitative analysis comparing four movement features of the results for all cost functions with the experimental data.
