# Supplementary material for: Optimality Principles in Human Point-to-Manifold Reaching Accounting for Muscle Dynamics
Source: Front Comput Neurosci. 2020 May 15;14:38. doi: 10.3389/fncom.2020.00038 (PMC7242656; doi:10.3389/fncom.2020.00038)
Supplement: Supplementary file 1 [file Data_Sheet_1.PDF]

Supplementary material describing Arm26

K. Stollenmaier, D. Haeufle

August 19, 2019

The neuro-musculo-skeletal model *Arm26* consists of a musculoskeletal model of the arm with 2 degrees of freedom actuated by six muscles and a controller. The model is implemented in C/C++ code within our in-house multi-body simulation code *demoa*. For a better overview, the implementation of the model is divided into three parts: the mechanical part (representing the bone structure and the muscle routing), the actuation of this mechanical part (muscle-tendon structures) and the controller (nervous system) which provides the input to the actuation part.

1 Musculoskeletal model of the arm: Mechanics and Actuation

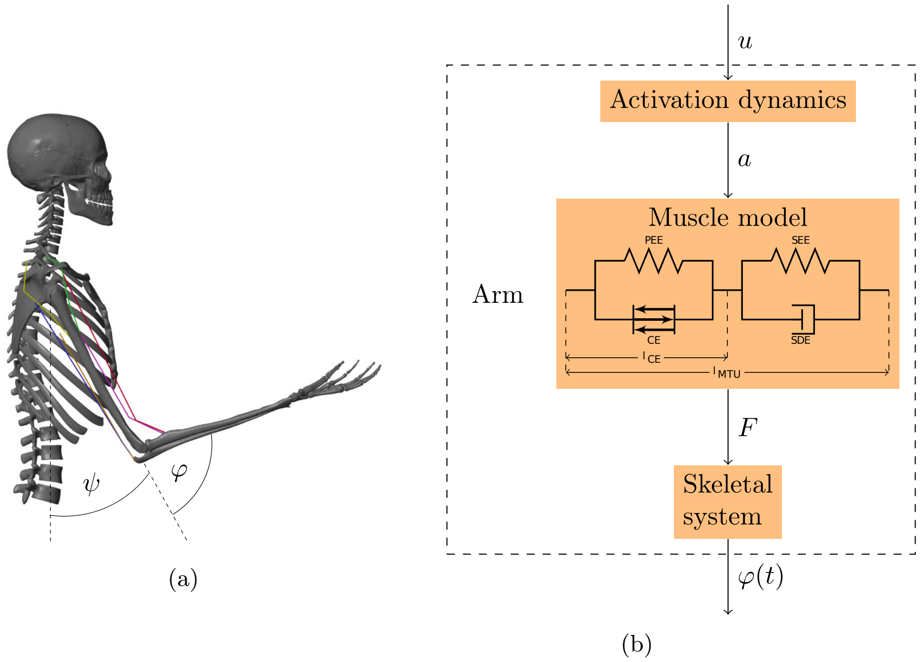

Figure 1: (a) Visualization of the musculoskeletal model of the arm and the definition of the shoulder angle ψ and the elbow angle φ and (b) Structure of the arm model: the motor command u is fed into the model of the activation dynamics of muscles which relates the neuronal stimulation to muscular activity a that drives the muscle model. The muscles produce forces F that act on the skeletal system resulting in a simulated movement $\varphi(t)$ of the arm.

The musculoskeletal model *Arm26* of the human arm uses the same geometry and muscle parameters as the simulation model described in Driess et al. (2018) which is based on Bayer et al. (2017). It consists of two rigid bodies (lower and upper arm) that are connected via two one-degree-of-freedom revolute joints that represent the shoulder and elbow joint. This multibody system is actuated by six muscle-tendon units (MTUs), four monoarticular and two biarticular muscles (see Figure 1a). The muscles are modeled as lumped muscles, i.e. they represent a multitude of anatomical muscles:

1. Monoarticular Elbow Flexor: *m. brachioradialis, m. brachialis, m. pronator teres, m. extensor carpi radialis*

2. Monoarticular Elbow Extensor: *m. triceps lateralis, m.triceps medialis, m. an-coneus, m. extensor carpi ulnaris*

3. Biarticular Elbow Flexor Shoulder Anteversion: *m. biceps brachii caput longum and caput breve*

4. Biarticular Elbow Extensor Shoulder Retroversion: *m. triceps brachii caput longum*

5. Monoarticular Shoulder Anteversion: *m. deltoideus (pars clavicularis, anterior, lateral), m. superior pectoralis major, m. coracobrachialis*

6. Monoarticular Shoulder Retroversion: *m. deltoideus (pars spinalis, posterior), m. latissimus dorsi*

The MTU structure is modeled using an extended Hill-type muscle model as described in Haeufle et al. (2014) with muscle activation dynamics as introduced by Hatze (1977). The muscle model is a macroscopic model consisting of four elements: the contractile element (CE), the parallel elastic element (PEE) and the serial elastic element (SEE) and serial damping element (SDE), as illustrated in Figure 1b. The inputs to the muscle model are the length of the muscle-tendon unit (MTU) l^{MTU}, the contraction velocity of the MTU $\dot{l}^{\mathrm{MTU}}$ and the muscular activity a. The output of the muscle model is a one-dimensional muscle force F^{MTU}. This force drives the movement of the skeletal system. For the routing of the muscle path around the joints, deflection ellipses are implemented as described by Hammer et al. (2019) (see Figure 2). The muscle path can move within these ellipses and is deflected as soon as it touches the boundary.

All in all, the governing model dependencies for all muscles $i = 1, ..., n$ are:

$$\begin{aligned} \dot{l}_i^{\mathrm{CE}} &= f_{\mathrm{CE}}(l_i^{\mathrm{CE}}, l_i^{\mathrm{MTU}}, \dot{l}_i^{\mathrm{MTU}}, a_i) && (1)\\ \dot{a}_i &= f_a(a_i, u_i, l_i^{\mathrm{CE}}) && (2)\\ F_i^{\mathrm{MTU}} &= f_F(l_i^{\mathrm{MTU}}, \dot{l}_i^{\mathrm{MTU}}, l_i^{\mathrm{CE}}, a_i) && (3)\\ \ddot{\mathbf{q}} &= f_q(\dot{\mathbf{q}}, \mathbf{q}, \mathbf{F}^{\mathrm{MTU}}), && (4) \end{aligned}$$

where $\mathbf{q}$ denotes a generalized state vector, in this case it can be defined as $\mathbf{q} = [\varphi, \psi]$ and $\mathbf{F}^{\mathrm{MTU}} = \{F_i^{\mathrm{MTU}}\}_{i=1}^{n}$.

The mechanical parameters of the arm segments are taken from Kistemaker et al. (2006) and can be found in Table 1. The positions and sizes of the deflection ellipses were chosen in order to match moment arms in literature. For more details on this see Suissa (2017). The (non-)muscle-specific parameters can be found in Table 2 and Table 3.

	Length [m]	d [m]	Mass [kg]	I [kgms^2]
Upper arm	0.335	0.146	2.10	0.024
Lower arm	0.263	0.179	1.65	0.025

Table 1: Mechanical parameters of the skeletal structure (Kistemaker et al. (2006)) with d=distance from proximal joint to center of mass and I=moment of inertia with respect to the center of mass.

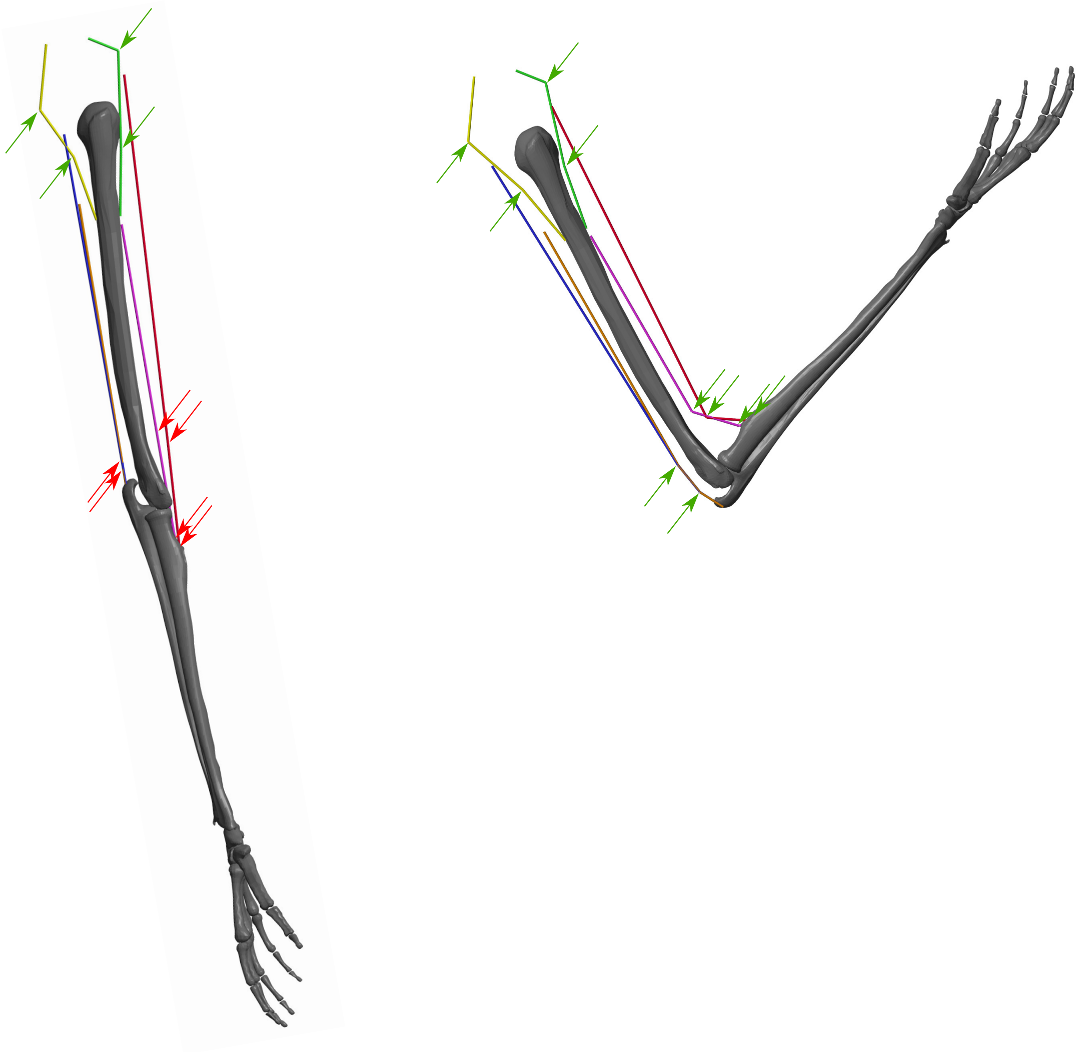

Figure 2: Illustration of the deflection ellipses that are used for the muscle routing in two different arm positions. Green arrows indicate active ellipses that deflect the muscle path, while red arrows indicate inactive ellipses that do not change the muscle path.

	F_{max} [N]	$l_{\text{CE,opt}}$ [m]	$l_{\text{SEE,0}}$ [m]
Monoarticular Elbow Flexor	1420	0.101	0.188
Monoarticular Elbow Extensor	1550	0.093	0.187
Monoarticular Shoulder Anteversion	838	0.134	0.039
Monoarticular Shoulder Retroversion	1207	0.140	0.066
Biarticular Elbow Flexor Shoulder Anteversion	414	0.159	0.236
Biarticular Elbow Extensor Shoulder Retroversion	603	0.141	0.240

Table 2: Muscle-specific actuation parameters (Kistemaker et al. (2006) and Kistemaker et al. (2013)), the lengths of $l_{\text{CE,opt}}$ and $l_{\text{SEE,0}}$ were adapted to match the muscle path routed through the ellipses in order to allow for a big range of motion. For this parameter adaptation method see Suissa (2017).

	Parameter	Unit	Value	Source	Description
CE	ΔW_{des}	[ ]	0.525	similar to Bayer et al. (2017); Kistemaker et al. (2006)	width of normalized bell curve in descending branch, adapted to match observed force-length curves
	ΔW_{asc}	[ ]	0.525	similar to Bayer et al. (2017); Kistemaker et al. (2006)	width of normalized bell curve in asscending branch, adapted to match observed force-length curve
	$\nu_{\mathrm{CE,des}}$	[ ]	1.5	Mörl et al. (2012)	exponent for descending branch
	$\nu_{\mathrm{CE,asc}}$	[ ]	3.0	Mörl et al. (2012)	exponent for asscending branch
	$A_{\mathrm{rel,0}}$	[ ]	0.2	Günther (1997)	parameter for contraction dynamics: maximum value of A_{rel}
	$B_{\mathrm{rel,0}}$	[1/s]	2.0	Günther (1997)	parameter for contraction dynamics: maximum value of B_{rel}
	$\mathcal{S}_{\mathrm{ecc}}$	[ ]	2.0	van Soest et al. (1993)	relation between $F(v)$ slopes at $v_{\mathrm{CE}} = 0$
	$\mathcal{F}_{\mathrm{ecc}}$	[ ]	1.5	van Soest et al. (1993)	factor by which the force can exceed F_{isom} for large eccentric velocities
PEE	$\mathcal{L}_{\mathrm{PEE,0}}$	[ ]	0.95	Günther (1997)	rest length of PEE normalized to optimal length of CE
	ν_{PEE}	[ ]	2.5	Mörl et al. (2012)	exponent of F_{PEE}
	$\mathcal{F}_{\mathrm{PEE}}$	[ ]	2.0	Mörl et al. (2012)	force of PEE if l_{CE} is stretched to ΔW_{des}
SDE	D_{SDE}	[ ]	0.3	Mörl et al. (2012)	dimensionless factor to scale $d_{\mathrm{SDE,max}}$
	R_{SDE}	[ ]	0.01	Mörl et al. (2012)	minimum value of d_{SDE} (at $F_{MTU} = 0$), normalized to $d_{\mathrm{SDE,max}}$
SEE	$\Delta U_{\mathrm{SEE,nll}}$	[ ]	0.0425	Mörl et al. (2012)	relative stretch at non-linear linear transition
	$\Delta U_{\mathrm{SEE,l}}$	[ ]	0.017	Mörl et al. (2012)	relativ additional stretch in the linear part providing a force increase of $\Delta F_{\mathrm{SEE,0}}$
	$\Delta F_{\mathrm{SEE,0}}$	[N]	$F_{\mathrm{max}} * 0.4$		both force at the transition and force increase in the linear part
Hatze	m	[1/s]	11.3	Kistemaker et al. (2006)	time constant for the activation dynamics
	c	[mol/l]	1.37e-4	Kistemaker et al. (2006)	constant for the activation dynamics
	η	[l/mol]	5.27e4	Kistemaker et al. (2006)	constant for the activation dynamics
	k	[ ]	2.9	Kistemaker et al. (2006)	constant for the activation dynamics
	q_0	[ ]	0.005	Günther (1997)	resting active state for all activated muscle fibers
	ν	[ ]	3	Kistemaker et al. (2006)	constant for the activation dynamics

Table 3: Muscle non-specific actuation parameters for the muscles and the activation dynamics.

References

Bayer, A., Schmitt, S., Günther, M., and Haeufle, D. F. The influence of biophysical muscle properties on simulating fast human arm movements. *Computer Methods in Biomechanics and Biomedical Engineering*, 20(8):803–821, 2017. doi: 10.1080/10255842.2017.1293663.

Driess, D., Zimmermann, H., Wolfen, S., Suissa, D., Haeufle, D., Hennes, D., Toussaint, M., and Schmitt, S. Learning to Control Redundant Musculoskeletal Systems with Neural Networks and SQP: Exploiting Muscle Properties. *ICRA 2018 (accepted)*, 2018.

Günther, M. *Computersimulationen zur Synthetisierung des muskulär erzeugten menschlichen Gehens unter Verwendung eines biomechanischen Mehrkörpermodells.* PhD thesis, Eberhard-Karls-Universität zu Tübingen, 1997.

Haeufle, D. F. B., Günther, M., Bayer, A., and Schmitt, S. Hill-type muscle model with serial damping and eccentric force-velocity relation. *Journal of Biomechanics*, 47(6):1531–1536, 2014. doi: 10.1016/j.jbiomech.2014.02.009.

Hammer, M., Günther, M., Haeufle, D., and Schmitt, S. Tailoring anatomical muscle paths: a sheath-like solution for muscle routing in musculo-skeletal computer models. *Mathematical Biosciences*, accepted (February):68–81, 2019. doi: 10.1016/j.mbs.2019.02.004.

Hatze, H. A Myocybernetic Control Model of Skeletal Muscle. *Biol. Cybernetics*, 25:103–119, 1977.

Kistemaker, D. a., Van Soest, A. J., and Bobbert, M. F. Is equilibrium point control feasible for fast goal-directed single-joint movements? *Journal of Neurophysiology*, 95(5):2898–912, 2006. doi: 10.1152/jn.00983.2005.

Kistemaker, D. A., Van Soest, A. J. K., Wong, J. D., Kurtzer, I., and Gribble, P. L. Control of position and movement is simplified by combined muscle spindle and Golgi tendon organ feedback. *Journal of Neurophysiology*, 109(4):1126–1139, 2013. doi: 10.1152/jn.00751.2012.

Mörl, F., Siebert, T., Schmitt, S., Blickhan, R., and Günther, M. Electro-Mechanical Delay in Hill-Type Muscle Models. *Journal of Mechanics in Medicine and Biology*, 12(05):1250085, 2012. doi: 10.1142/s0219519412500856.

Suissa, D. R. *Modeling , Control and Optimization in Human Motor Control : A Simulation Study of a Physiological Human Arm.* Master thesis, University of Stuttgart, 2017.

van Soest, A., Bobbert, M., Iijima, T., Shimizu, K., and Asanuma, N. The Contribution of Muscle Properise in the Control of Explosive Movments. *Biological Cybernetics*, 69(3):195–204, 1993.
