# Supplementary material for: Optimality Principles in Human Point-to-Manifold Reaching Accounting for Muscle Dynamics
Source: Front Comput Neurosci. 2020 May 15;14:38. doi: 10.3389/fncom.2020.00038 (PMC7242656; doi:10.3389/fncom.2020.00038)
Supplement: Supplementary file 2 [file Data_Sheet_2.pdf]

Supplementary Material: Optimality principles in human point-to-manifold reaching accounting for muscle dynamics

Isabell Wochner [1], Danny Driess [2], Heiko Zimmermann [3], Daniel F. B. Haeufle [4], Marc Toussaint [2], and Syn Schmitt [1]

[1] *Institute for Modelling and Simulation of Biomechanical Systems, University of Stuttgart, Stuttgart, Germany,* [2] *Machine Learning and Robotics Lab, University of Stuttgart, Stuttgart, Germany,* [3] *Khoury College of Computer Sciences, Northeastern University, Boston, MA, United States,* [4] *Hertie Institute for Clinical Brain Research, and Werner Reichard Centre for Integrative Neuroscience, University of Tübingen,Tübingen, Germany*

1 POINT-TO-POINT SIMULATIONS

Point-to-point simulations were conducted similar to the point-to-manifold simulations, as described in Section 2. As target point we set $x^\star = (0.5083, -0.25)\,\mathrm{m}$. We accepted an error tolerance of $3.6\,\mathrm{cm}$, which was chosen because the original point-to-manifold experiments (as reported in Berret et al. (2011)) showed an error of $2.2 \pm 1.4\,\mathrm{cm}$ on average across subjects without visual feedback. The x-coordinate is the same as for the point-to-manifold task and corresponds to 85% of the full arm length of the model, and the z-coordinate was selected as the endpoint from the best simulated trajectory of the J_{JEE} cost function. Again, our neuro-musculo-skeletal model predicts eight different trajectories, one for each optimality principle. Figure S1 (a) shows the simulated trajectories corresponding to the best u of each cost function, which were found using Bayesian optimization. Additionally, the target point $x^\star$ with the radius of the acceptable error tolerance of $3.6\,\mathrm{cm}$ is plotted. The right subplot (Fig. S1 (b)) shows the corresponding predicted tangential velocities for each of the eight different cost functions. As shown in Figure S1 almost all predicted trajectories for the point-to-point movement show roughly straight paths and bell-shaped speed profiles similar to previous findings in the literature (e.g. Abend et al. (1982); Flash and Hogan (1985); Todorov (2004); Harris and Wolpert (1998)). This makes it almost impossible to decide which cost function is the true one based on the given task since they all have a good neurophysiological basis and predict very similar trajectories.

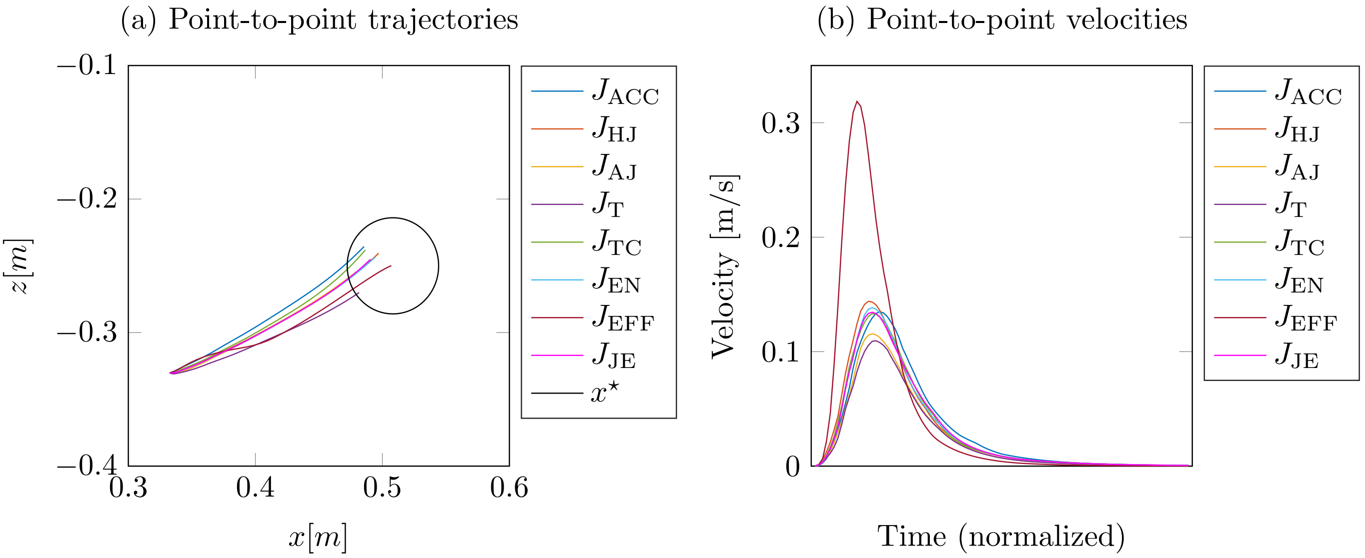

Figure S1. (a) The best predicted trajectories for the fingertip movement pointing to the target point $x^\star$ are shown. Eight different cost functions are compared. (b) The respective predicted tangential velocities for each of the eight different cost functions are shown.

REFERENCES

Abend, W., Bizzi, E., and Morasso, P. (1982). Human arm trajectory formation. *Brain: a journal of neurology* 105, 331–348

Berret, B., Chiovetto, E., Nori, F., and Pozzo, T. (2011). Evidence for composite cost functions in arm movement planning: an inverse optimal control approach. *PLoS computational biology* 7

Flash, T. and Hogan, N. (1985). The Coordination of Arm Movements: an experimentally confirmed mathematical model'. *Journal of Neuroscience* 5, 1688–1703

Harris, C. M. and Wolpert, D. M. (1998). Signal-dependent noise determines motor planning. *Nature* 394, 780

Todorov, E. (2004). Optimality principles in sensorimotor control. *Nature neuroscience* 7, 907–915. doi:10.1038/nn1309
