# Supplementary material for: Optimality Principles in Human Point-to-Manifold Reaching Accounting for Muscle Dynamics
Source: Front Comput Neurosci. 2020 May 15;14:38. doi: 10.3389/fncom.2020.00038 (PMC7242656; doi:10.3389/fncom.2020.00038)
Supplement: Supplementary file 3 [file Data_Sheet_3.pdf]

Supplementary Material: Optimality principles in Human Point-to-Manifold Reaching Accounting for Muscle Dynamics

Isabell Wochner [1], Danny Driess [2], Heiko Zimmermann [3], Daniel F. B. Haeufle [4], Marc Toussaint [2], and Syn Schmitt [1]

[1] *Institute for Modelling and Simulation of Biomechanical Systems, University of Stuttgart, Stuttgart, Germany,* [2] *Machine Learning and Robotics Lab, University of Stuttgart, Stuttgart, Germany,* [3] *Khoury College of Computer Sciences, Northeastern University, Boston, MA, United States,* [4] *Hertie Institute for Clinical Brain Research, and Werner Reichard Centre for Integrative Neuroscience, University of Tübingen,Tübingen, Germany*

1 QUANTITATIVE ANALYSIS OF PREDICTED TRAJECTORIES AND VELOCITIES

It is still an open question in motor control which metric to use to compare movements with different trajectories and different velocity profiles (Gielen, 2009; Berret et al., 2011). To overcome this issue, we selected four major movement features and compared them quantitatively in the following. Note that we compared the simulated data to the average experimental data. It might be interesting to compare the simulated data also with one specific trajectory measured from an individual which has the same anatomical and mechanical parameters as our model (e.g. segment lengths, maximum muscle force, moment arms); however, this data is not available in this case for investigating optimality principles.

We compared the following quantities: endpoint error, signed curvature error, peak velocity and skewness. The reasons for this choice are the following: For point-to-point movements, it is typically reported that probands show roughly straight curves and bell-shaped velocity profiles (e.g. Abend et al. (1982); Flash and Hogan (1985); Harris and Wolpert (1998); Todorov (2004)). Straightness of curves can be measured using the signed curvature error, as discussed in Section 1.2. To determine the bell-shapedness of the velocity profiles, the skewness of the tangential velocity can be measured as described in 1.4. Another standard kinematic parameter which was used in previous arm pointing experiments (Ben-Itzhak and Karniel, 2008; Berret et al., 2011; Hilt et al., 2016) is the peak velocity (see Sect. 1.3). Further, we included the endpoint error on the bar (see Sect. 1.1). This quantity is especially interesting in the point-to-manifold task compared to the point-to-point task, as the target point on the bar is not set a priori.

First, the absolute values for all movement features were calculated, which are shown in Table S1. For the experimental data, the average value and the standard deviation is given. Second, the (absolute) difference between the predicted value of each cost function with the averaged experimental value was calculated and is shown in Figure S2.

1.1 Endpoint

As the first movement feature, we compared the endpoints of each trajectory to the averaged endpoint of the experimental data. Note, that only the z-coordinate is given because the x-coordinate is the same for all compared trajectories, namely equal to the position of the bar. As shown in Figure S2 (a), the error of the

predicted endpoint of the J_{JE} cost function and of the final proposed cost function J_{JEE} (restricted to 1 sec) in comparison to the experimental mean endpoint, is the smallest. Furthermore, it is shown (both in Tab. S1 and in Fig. S2 (a)) that the four cost functions J_{ACC}, J_{HJ}, J_{AJ} and J_{TC} have an higher endpoint on the bar, compared to the experimental data, whereas the three cost functions J_{T}, J_{EN}, and J_{EFF} have a lower endpoint on the bar.

1.2 Signed Curvature Error

To determine, whether the curvature of the predicted trajectories is convex, concave or straight, we calculated the signed curvature error. It is defined as the maximum deviation from the line connecting the start and endpoint of the trajectory. Therefore, a positive sign corresponds to a concave trajectory, whereas a negative sign denotes a convex trajectory. As shown in Table S1, only the J_{JE} and the J_{JEE} cost function have a positive signed curvature error, similar to the experimental data. All other cost functions show either a concave behavior (negative sign) or predict almost a straight line (signed curvate error almost zero) as in the case of the J_{T} cost function. Furthermore, the absolute deviation of the curvature error between the experimental trajectory and each cost function is shown in Fig. S2 (b). The deviation of the signed curvature error between the J_{JEE} cost function and the experimental value is the smallest.

1.3 Peak Velocity

As third movement feature, we determined the peak velocity. Again, the absolute values are given in Tab. S1, in the fourth column. All predicted peak velocities are smaller than the averaged peak velocity of the experimental data. However, the peak velocity error of the J_{JEE} (restricted to 1 sec) has the smallest error and is the closest to the experimental data as visualized in Fig. S2 (c).

1.4 Skewness

For point-to-point movements, it is typically reported that probands show bell-shaped velocity profiles. To determine whether the profile is bell-shaped or left- or right-skewed, we calculated the skewness S of the velocity curves as follows:

$$S = \sum_{i=1}^{n} \tilde{v} \left(\frac{t_i - \mu(\tilde{v})}{\sqrt{\sigma(\tilde{v})}} \right)^3 \tag{S1}$$

Here, n denotes the length of the considered quantity, t the time, μ the mean value and σ the variance. Furthermore, the normalized velocity $\tilde{v}$ is defined as:

$$\tilde{v} = \frac{v(t)}{\sum(v(t))} \tag{S2}$$

Positive values for S denote a right-skewed curve, negative values stand for left-skewness, and values close to zero correspond to bell-shaped curves. The absolute skewness values for each cost function and the experimental mean is given in the last column in Tab. S1. Both the experimental mean, as well as the J_{JEE} skewness value, are close to zero, which stands for bell-shaped velocity profiles. All other predicted values are positive, which means that they show a right-skewness. The deviation in skewness between the experimental data and the predicted values is shown in Fig. S2 (d).

Evaluated Trajectory	Endpoint $[m]$	Signed Curvature $[m]$	Peak Velocity $[m/s]$	Skewness
J_{ACC}	-0.111	-0.027	0.192	0.985
J_{HJ}	-0.099	-0.021	0.252	0.997
J_{AJ}	-0.123	-0.015	0.573	1.194
J_{T}	-0.329	-0.005	0.333	1.146
J_{TC}	-0.018	-0.033	0.255	0.998
J_{EN}	-0.295	-0.035	0.346	1.127
J_{EFF}	-0.433	-0.145	0.567	0.771
J_{JE}	-0.185	0.032	0.205	1.099
J_{JEE}	-0.249	0.011	0.847	0.285
experimental data	-0.212 ± 0.084	0.012 ± 0.012	0.8721 ± 0.21	0.337 ± 0.112

Table S1. Quantitative Analysis of cost functions in comparison with experimental data

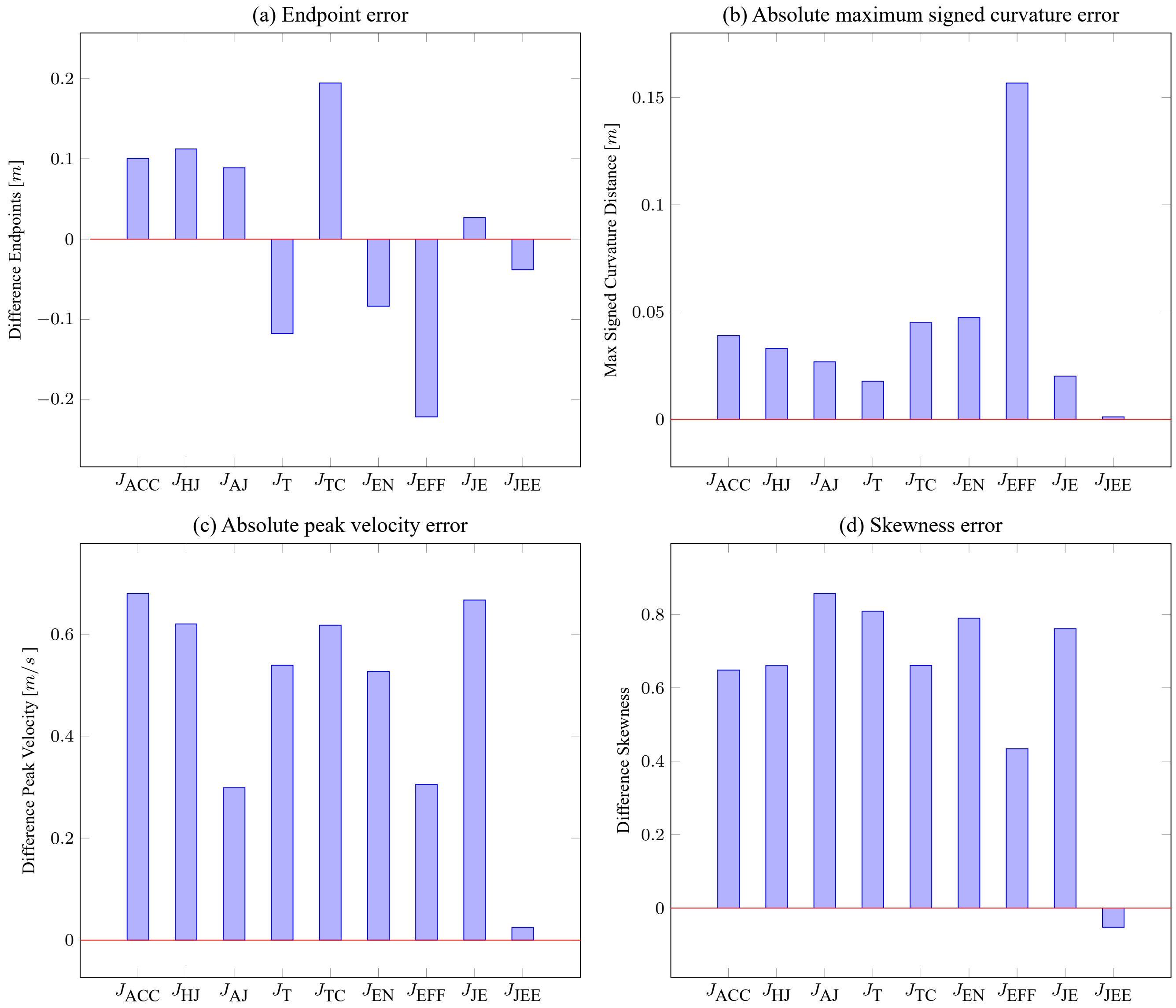

Figure S2. Quantitative Analysis of the results for the different cost functions in comparison with the experimental data is shown. We compared the four movement features: endpoint error on the bar, maximum signed curvature error, peak velocity, and skewness. The blue bars show the deviation between the predicted quantity and the experimental quantity. The red line represents the baseline corresponding to the experimental reference value.

REFERENCES

Abend, W., Bizzi, E., and Morasso, P. (1982). Human arm trajectory formation. *Brain: a journal of neurology* 105, 331–348

Ben-Itzhak, S. and Karniel, A. (2008). Minimum acceleration criterion with constraints implies bang-bang control as an underlying principle for optimal trajectories of arm reaching movements. *Neural Computation* 20, 779–812

Berret, B., Chiovetto, E., Nori, F., and Pozzo, T. (2011). Evidence for composite cost functions in arm movement planning: an inverse optimal control approach. *PLoS computational biology* 7

Flash, T. and Hogan, N. (1985). The Coordination of Arm Movements: an experimentally confirmed mathematical model'. *Journal of Neuroscience* 5, 1688–1703

Gielen, S. (2009). Review of models for the generation of multi-joint movements in 3-D. In *Progress in Motor Control* (Springer). 523–550

Harris, C. M. and Wolpert, D. M. (1998). Signal-dependent noise determines motor planning. *Nature* 394, 780

Hilt, P., Berret, B., Papaxanthis, C., Stapley, P. J., and Pozzo, T. (2016). Evidence for subjective values guiding posture and movement coordination in a free-endpoint whole-body reaching task. *Scientific reports* 6, 23868

Todorov, E. (2004). Optimality principles in sensorimotor control. *Nature neuroscience* 7, 907–915. doi:10.1038/nn1309
